# Three-dimensional CT angiography facilitates uniportal thoracoscopic anatomic lung resection for pulmonary sequestration: a retrospective cohort study

**DOI:** 10.1186/s13019-022-01975-8

**Published:** 2022-08-30

**Authors:** Wenlong Zheng, Miao Zhang, Wenbin Wu, Hui Zhang, Xinhui Zhang

**Affiliations:** 1 Department of General Surgery, Xuzhou Central Hospital, Affiliated Xuzhou Clinical College of Xuzhou Medical University, 199 Jiefang South Road, Xuzhou, 221009 China; 2 Department of Thoracic Surgery, Xuzhou Central Hospital, Affiliated Xuzhou Clinical College of Xuzhou Medical University, 199 Jiefang South Road, Xuzhou, 221009 China

**Keywords:** Bronchopulmonary sequestration (BPS), Pulmonary sequestration (PS), Video-assisted thoracoscopic surgery (VATS), Three-dimensional computed tomography angiography (3D-CTA)

## Abstract

**Background:**

Pulmonary sequestration (PS) is a rare lesion with independent blood supply from an anomalous systemic artery. A timely resection is considered as the best treatment for PS. Three-dimensional computed tomography angiography (3D-CTA) has been widely utilized for precise thoracic surgery. This study aimed to investigate the role of preoperative 3D-CTA and resection simulation in uniportal video-assisted thoracoscopic surgery (VATS) anatomical lung surgery for PS.

**Methods:**

The data of 20 consecutive PS patents undergoing anatomic lung resection between April 2011 and May 2021 in a single centre were retrospectively reviewed. These patients were divided into the 3D-CTA group (10 patients) and the control group (10 patients) according to the initial surgical planning with or without 3D-CTA. The perioperative parameters regarding safety and fluency such as the incidence of conversion to open thoracotomy, operation time, blood loss, complications and chest tube duration were analyzed.

**Results:**

This cohort included 12 female and 8 male patients, with a mean age of 45 years old (range 24–60 years). Nine cases demonstrated recurrent febrile, cough, or hemoptysis; whereas the other 11 patients were asymptomatic on admission. Eighteen (90.0%) intralobar and 2 extralobar PS were confirmed, whereas 18 (90.0%) lesions were located in the left thorax. The feeding vessels originated from the thoracic aorta in 16 patients (80.0%), the abdominal aorta in 3 (15.0%), and the inferior phrenic artery in 1 patient (5.0%). There was no major bleeding or 30 days mortality. The initial surgical planning included 9 uniportal and 1 two-port VATS in the 3D-CTA group, as compared with 10 two-port VATS in the control group. Thirteen lobectomies, 5 segmentectomies and 2 mass excisions were performed. However, no conversion was needed in the 3D-CTA group; whereas 6 (60.0%) conversions (4 to multiple-port and 2 to thoracotomy) occurred in the control group, indicating a significant difference (*P* = 0.003). In addition, the operation time in the 3D-CTA group was significantly shorter than those in the control group [(108.5 ± 24.9) min vs. (154.5 ± 39.4) min, *P* = 0.006]. The other surgery-related outcomes were similar between the two groups.

**Conclusion:**

Preoperative 3D-CTA facilitates the safe and fluent performance of uniportal VATS anatomical lung resection for PS with shortened operation time and lessened surgical conversions.

## Background

Pulmonary sequestration (PS) is classified into intralobar sequestration (ILS) and extralobar sequestration (ELS), with a non-functioning abnormal lung tissue receiving arterial supply by one or more systemic vessels. The best treatment choice is a timely video-assisted thoracoscopic surgery (VATS) lobectomy or segmentectomy to remove the sequestrated lung, regardless of the symptoms. Two-dimensional multi-detector computed tomography (MDCT) plays a key role in the diagnosis of PS and preoperative surgical planning. PS has a variety of appearances in CT images including consolidation, mass, or air/fluid-filled cystic or multicystic lesion [1].

In fact, the experience of the radiologists and the surgeons play an important role in correct diagnosis of PS, because the differential diagnosis of PS, atelectasis, and intra-thoracic tumor is sometimes difficult. Once the feeding vessels into the pulmonary mass were indicated, a PS is almost diagnosed exclusively. However, for inexperienced surgeons, a two-dimensional vision of the PS is always insufficient to accurately and definitely identify the number and location of the aberrant effluent and influent vessel(s) during preoperative simulation. In addition to the confirmation of resection range of the sequestrated lung, the precise verification of the abnormal PS vessels is critical to facilitate the safe and fluent performance of PS resection. Three-dimensional CT angiography (3D-CTA) digital anatomical models were therefore utilized to definitely identify the feeding arteries with or without the accompanied drainage veins of the PS. The first case of uniportal VATS lobectomy for intralobar PS was reported in 2015, assisted with 3D-CT reconstruction [2]. Herein a retrospective cohort study was conducted to summarize the mid-term outcomes and potential advantages of uniportal VATS anatomical lung resection for PS patients assisted with preoperative 3D-CTA versus those without 3D models.

## Patients and methods

### Patients

The clinical data of 20 consecutive patients undergoing anatomical lung resection for PS in Xuzhou Central Hospital between April 2011 and May 2021 were retrospectively reviewed. They were categorized into the 3D-CTA group and the control group according to preoperative surgical planning with or without 3D-CTA digital anatomy, with 10 patients in each group. Baseline demographics (age, gender, body mass index, pulmonary lung function), the characteristics of the PS (location, subtype, origin of the aberrant arteries), and the perioperative variables (operation time, estimated blood loss, complications, and duration of chest drainage) were recorded (Tables 1 and 2). There were 12 female patients (60.0%) in this cohort, and the mean age of the patients was 45 years (range 23–60 years). In total, there patients indicated 18 (90.0%) ILS and 2 (10.0%) ELS. In addition, 2 (10.0%) PS were located in the right lower lobe and 18 (90.0%) were in the left thorax. Meanwhile, 16 (80.0%), 3 (15.0%) and 1 (5.0%) PS showed aberrant feeding vessels originated from the thoracic aorta, abdominal aorta, and inferior phrenic artery, respectively. Moreover, the major symptoms were recurrent cough, expectoration or fever (5 cases, 25.0%), and hemoptysis (4 case, 20.0%); whereas the other 11 (55.5%) patients were asymptomatic on admission (Table 1). Patients with respiratory symptoms were controlled using antibiotics for the safety of surgery to diminish the potential risk of bronchopleural fistula. All the patients revealed a single aberrant feeding artery, and they demonstrated normal cardiopulmonary function before surgery. Previous chest surgery, and estimated dense pleural adhesions as shown in the CT images were not considered as contraindications of uniportal VATS. Actually, intent-to-treat protocol was used in this retrospective cohort study.

**Table 1:** Baseline characteristics of the patients with pulmonary sequestration

Variables	Total (n = 20)	3D-CTA group (n = 10)	Control group (n = 10)	*P* value
Age (range), y	45.0 ± 9.1 (23–60)	40.6 ± 12.0 (23–60)	44.7 ± 8.4 (26–55)	0.389
Female, n (%)	12 (%)	6 (60)	6 (60)	1.000
Body mass index (range), kg/m^2^	24.6 ± 3.0 (19.6–29.4)	25.2 ± 3.0 (20.2–29.4)	23.3 ± 3.1 (19.6–27.7)	0.181
Major symptoms on admission, n (%)				0.079
Recurrent respiratory tract infection (cough/expectoration/fever)	5 (25.0)	4 (40.0)	1 (10.0)	
Hemoptysis	4 (20.0)	3 (30.0)	1 (10.0)	
Asymptomatic	11 (55.0)	3 (30.0)	8 (80.0)	
Sequestration type, n (%)				1.000
Intralobar	18 (90.0)	9 (90.0)	9 (90.0)	
Extralobar	2 (10.0)	1 (10.0)	1 (10.0)	
Location of the sequestrated lung, n (%)				0.136
Left lower lobe	18 (90.0)	8 (80.0)	10 (100)	
Right lower lobe	2 (10.0)	2 (20.0)	0	
Origin of the aberrant feeding vessels, n (%)				0.119
Thoracic aorta	16 (80.0)	7 (70.0)	9 (90.0)	
Abdominal aorta	3 (15.0)	3 (30.0)	0	
Inferior phrenic artery	1 (5.0)	0	1 (10.0)	
Venous drainage of the sequestrated lung, n (%)				0.361
Pulmonary veins	8 (40.0)	3 (30.0)	5 (50.0)	
Undetermined	12 (60.0)	7 (70.0)	5 (50.0)	
Preoperative FEV1 (range), L	3.0 ± 0.5 (1.8–3.8)	2.9 ± 0.6 (1.9–3.6)	2.9 ± 0.6 (1.8–3.8)	0.856

*3D-CTA* three-dimensional computed tomographic angiography, *FEV1* forced expiratory volume in one second

**Table 2:** Perioperative data of the patients

Variables	Total (n = 20)	3D-CTA group (n = 10)	Control group (n = 10)	*P* value
Extensive pleural adhesion, n (%)	5 (25.0)	3 (30.0)	2 (20.0)	0.605
Surgical procedures, n (%)				0.144
Lobectomy	13 (65.0)	6 (60.0)	7 (70.0)	
Segmentectomy	5 (25.0)	4 (40.0)	1 (10.0)	
Mass resection	2 (10.0)	0	2 (20.0)	
Initial surgical planning, n (%)				< 0.001*
Uniportal VATS	9 (45.0)	9 (90)	0	
Two-port VATS	11 (55.0)	1 (10)	10 (100)	
Conversion of the surgical approach, n (%)	6 (30.0)	0	6 (60.0)	0.003*
Three-port VATS	4 (20.0)	0	4 (40.0)	
Thoracotomy	2 (10.0)	0	2 (20.0)	
Disconnection of aberrant vessels, n (%)				0.531
Endo-stapler	17 (85.0)	9 (90.0)	8 (80.0)	
Ligation using silk	3 (15.0)	1 (10.0)	2 (20.0)	
Mean operation time (range), min	131.5 ± 39.8 (75–220)	108.5 ± 24.9 (75–150)	154.5 ± 39.4 (100–220)	0.006*
Estimated blood loss (range), mL	92.5 ± 40.6 (50–200)	85.0 ± 41.2 (50–150)	100.0 ± 40.8 (50–200)	0.424
Postoperative complications, n (%)	4 (20.0)	1 (10.0)	3 (30.0)	0.264
Pulmonary infection	1 (5.0)	0	1 (10.0)	
Prolonged air leakage (> 5 days)	1 (5.0)	1 (10.0)	0	
Chylothorax	1 (5.0)	0	1 (10.0)	
Bronchopleural fistula	1 (5.0)	0	1 (10.0)	
Duration of the chest tube (range), d	6.8 ± 2.8 (3–15)	7.8 ± 3.4 (3–13)	7.2 ± 3.3 (4–15)	0.485
Total thorax drainage volume (range), mL	1233.8 ± 771.8 (350–3150)	926.0 ± 450.0 (350–1850)	1541.5 ± 918.9 (550–3150)	0.073
Postoperative hospital stay (range), d	8.9 ± 3.9 (4–19)	8.0 ± 3.5 (4–16)	9.6 ± 4.2 (5–19)	0.312
Pathological diagnosis, n (%)				0.315
PS	19 (95.0)	9 (90.0)	10 (100)	
Carcinoma within the PS	1 (5.0)	1 (10.0)	0	
30-days mortality	0	0	0	–
Follow up (range), month	35.3 ± 19.0 (5–75)	32.6 ± 20.2 (5–75)	38.0 ± 18.3 (12–70)	0.539
Recurrence of cough or hemoptysis	0	0	0	–

*3D-CTA* three-dimensional computed tomographic angiography, *VATS* video-assisted thoracoscopic surgery, *PS* pulmonary sequestration

*P < 0.05

### Preoperative 3D-CTA and resection simulation

Initially, the PS was diagnosed by thin-slice two-dimensional MDCT images by the radiologists and the surgeons. Since the primary introduction of 3D-CT reconstruction technique in our hospital, the patients in this cohort established 3D-CTA by the free software, OsiriX (Apple Inc., California) using contrast-enhanced thin-slice MDCT sections (1.0 mm thickness) of the thorax and upper abdomen. The CTA images could definitely identify the diameter, length, and the course of the aberrant feeding vessels for surgical resection simulation. The initial surgical planning included 9 uniportal and 1 two-port VATS in the 3D-CTA group, as compared with 10 two-port VATS in the control group.

### Surgical technique

All patients were operated on under general anesthesia with selective one-lung ventilation using a double-lumen endotracheal tube.

For uniportal VATS, only an approximately 3.0–4.5 cm single incision was made at the 5th intercostal space of the anterior axillary line with a wound protector (Johnson, New Brunswick, NJ, USA), without rib-spreading. A 30°, 10 mm thoracoscope (Karl Storz, Tuttlingen, Germany) was utilized for vision. Anatomic lobectomy, segmentectomy or mass resection of the sequestrated lung was performed for ILS and ELS respectively, using the standard uniportal technique as reported [3]. First, the aberrant feeding vessels were meticulously ligated using a non-absorbable silk suture or clipped with 1–2 Hemo-o-Lock vascular clips (Weck Surgical Instruments, Teleflex Medical, Durham, NC); whereas the linear staplers Endo-GIA (Medtronic Inc., Shanghai, China) were used for the vessels with a diameter of larger than 6 mm after suture ligation to reduce the risk of bleeding [4]. Next, the anatomic lung resection was performed after the division of the feeding vessels. The lobar or segmental vein, artery and bronchus were divided gradually. Each specimen was placed in a sterilized storage bag and pulled out. One 20F chest tube was placed for pleural drainage.

For two-port VATS, an additional port was created along the 7th intercostal space at the midaxillary line while the working port was placed in the 4th or 5th intercostal space along the anterior axillary line.

In addition, the enhanced recovery after lung surgery protocol was utilized individually for the patients in this cohort. Early removal of the urine tube allowed patients to mobilize freely, which was helpful to diminish the risk of atelectasis and deep venous thrombosis. The drainage tube was removed when the drainage amount decreased to 200 mL per day without air leak or obvious residual pneumothorax as shown in the chest X-ray. Patients were usually discharged the day after the removal of the chest tube.

### Statistical analysis

The continuous variables were presented as means ± standard deviations and range due to the limited number of this cohort, whereas the categorical variables were presented as numbers and frequencies (%). Differences between uniportal and two-port VATS groups were determined using analysis of variance (ANOVA) for continuous variables and Fisher’s exact test for categorical variables. A P value of less than 0.05 was considered a significant difference. The data were analyzed using IBM SPSS Statistics for Windows, version 19.0 (IBM Corp., Armonk, NY, USA).

## Results

**Fig. 1:**
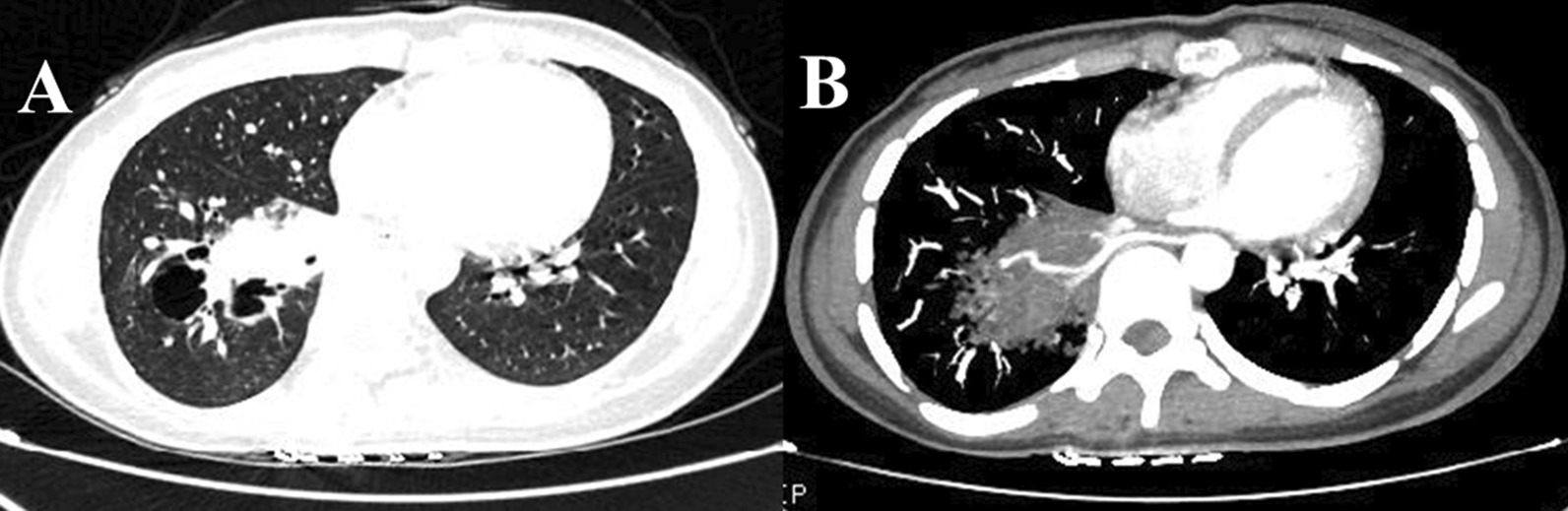
The sequestrated lung located in the right lower lobe was diagnosed using conventional two-dimensional computed tomography. **A** The lesion mimics bronchiectasis and infection; **B** contrast-enhanced images revealed the aberrant feeding vessel

**Fig. 2:**
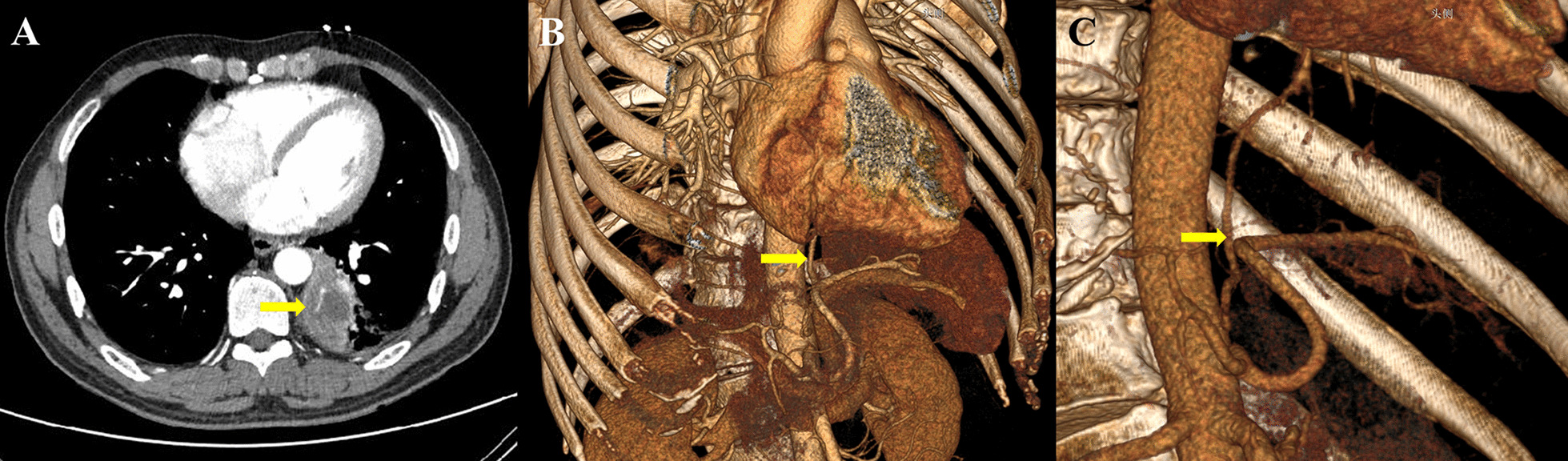
Three-dimensional computed tomography angiography showed definitely the anomalous blood supply. **A** A mass was located in the left lower lobe; **B** the abnormal feeding artery was next to the spleen artery; **C** the feeding artery originated from the abdominal aorta was finally confirmed

The first 10 patients in the control group were diagnosed as PS using thin-slice two-dimensional MDCT images before the introduction of 3D-reconstruction software (Fig. 1). Preoperative 3D-CTA digital anatomy models were established in the following 10 patients, which revealed definitely the abnormal blood vessels into the PS (Fig. 2). Most of the anomalous vessels could be identified around the lower pulmonary ligament.

Thirteen lobectomies and 5 segmentectomies were performed for ILS; whereas 2 mass resections were carried out for ELS. The perioperative features were shown in Table 2. There was no emergency reoperation or major bleeding in this cohort. Six (60.0%) conversions of the surgical procedure included 4 to multi-port VATS and 2 to thoracotomy were recorded in the control group, indicating an obviously higher incidence of conversion as compared with those in the 3D-CTA group (6 [60.0%] vs. 0, *P* = 0.003). Additionally, the operation time of the patents in the 3D-CTA group was significantly shorter than those in the control group ([108.5 ± 24.9] min vs. [154.5 ± 39.4] min, *P* = 0.006). However, the estimated blood loss ([85.0 ± 41.2] mL vs. [100.0 ± 40.8] mL), pleural drainage duration ([7.8 ± 3.4] d vs. [7.2 ± 3.3] d), total drainage volume ([926.0 ± 450.0] mL vs. [1541.5 ± 918.9] mL), and postoperative hospital stay ([8.0 ± 3.5] d vs. [9.6 ± 4.2] d) were similar between the 3D-CTA and the control groups (*P* > 0.05, respectively).

The postoperative course was mainly uneventful. The 3D-CTA group reported a (10.0%) case of prolonged air leak (> 5 days); whereas the control group showed 3 (30.0%) cases of complications (1 case of chylothorax, 1 case of bronchopleural fistula, and 1 case of pulmonary infection), without a statistical difference between the two groups (*P* = 0.264). All the complications were cured after conservative therapies. All the patients were discharged home in good condition after the removal of the chest tubes.

Pathological and immunohistochemical staining of the resected lung tissues confirmed the diagnosis of PS; whereas one specimen revealed neuroendocrine carcinoma arising within the PS. During the median follow-up of 35.3 months (range 5–75 months), no patients presented a recurrence of hemoptysis or dyspnea.

## Discussion

The present retrospective cohort study confirmed the obvious superiority, fluency and safety of uniportal VATS anatomic lobectomy and segmentectomy assisted with preoperative 3D-CTA, as indicated by the shortened operation time and the lessened incidence of surgical conversion of the patients in the 3D-CTA group. Meanwhile, the patients in the 3D-CTA group also demonstrated lessened intraoperative blood loss, total thorax drainage volume and shortened hospital stay after PS resection, although the difference was not statistically significant, probably due to the limited sample size. Preoperative confirmation of the aberrant vessels significantly facilitates the operation for PS, although it is not indispensable because careful dissection is mostly sufficient for experienced surgeons. There are several issued should be clarified accordingly.

To date, no established guidelines have been established for the treatment of PS. Endovascular occlusion of the feeding artery probably leads to the necrosis, fibrosis and progressive involution of the sequestrated lung and therefore to minimize the risk of intraoperative bleeding. Thus, embolization plus surgical resection might be the optimal approach for complex cases with multiple or large feeding vessels. In addition, the time of surgery for PS is still controversial. An early resection of the sequestrated lung during childhood may prevent the subsequent respiratory symptoms and to prevent the rare and debated risk of transformation into malignancy [5].

A substantial number of lung anomalies present incidental or recurrent respiratory infections progressing into adulthood. Encountering these lung anomalies in adults is a diagnostic challenge and they are often mistaken and followed by unnecessary procedures [6]. For ILS patients, the venous drainage is into the pulmonary vein. Approximately two-thirds of the ILS lesions are found incidentally in the posterior basal segment of the left lower lobe; whereas the PS associated with Scimitar syndrome (partial anomalous pulmonary venous connection) is more common in the right thorax [7]. For ELS patients, the venous drainage is drained into the systemic veins including the lower lobe vein, azygos vein, hemiazygos vein, subclavian or portal vein; whereas the arterial supply might come from the descending thoracic aorta (73%), the cranial portion of the abdominal aorta, celiac trunk, splenic artery (21%), intercostal arteries, pericardiophrenic, right coronary artery, subclavian, and internal thoracic arteries. Meanwhile, the ELS are often found between the diaphragm and the lower lobes [7]. Additionally, the number of the aberrant feeding artery into the PS may be more than one. Furthermore, the blood pressure of the aberrant arteries, which are often accompanied by inflammation and adhesion around the vessel, is sometimes high in PS patients. Moreover, PS should be distinguished from the anomalous systemic arterial supply into the lung, because they both showed independent aberrant artery but the treatment aim and procedures are different. Thus, the differential diagnosis is essential to avoid fatal postoperative complications [8].

Therefore, the major challenge during operation for PS is the identification of the aberrant vessels. Two-dimensional MDCT image is somewhat insufficient to provide a comprehensive assessment for complex PS, especially for inexperienced surgeons in a local hospital with small operation volume; whereas the preoperative identification of individualized drainage of the feeding vessels using a more practical tool such as 3D-CTA is essential for the safe and fluent performance of anatomic lung resection. Digital anatomy models established by 3D-CTA are therefore utilized to identify the variations of the PS in the present cohort study, which can help definitely confirm the diagnosis, overcome technical challenges, and improve the safety with fluency [9–11].

Uniportal VATS has evolved dramatically into a sophisticated procedure capable of performing major complex lung resection [12]. A meta-analysis shows that the perioperative outcomes of uniportal and multiport VATS anatomic pulmonary resection are equivalent [13]; however, another systematic review reports that it remains premature to declare its superiority [14]. Due to the limited sample size of the present cohort study, the quantitative analysis might deliver misleading information. Therefore, a thorough online literature review was conducted. We searched PubMed, Web of Science, Scopus, Embase, Cochrane Library and Google Scholar for relevant English reports of VATS lung resection for PS up to September 24th, 2021 based on the population, intervention, comparator, outcome and strategy (PICOS) framework. Key words and MeSH terms in title or abstract including “Video-Assisted”, “Thoracoscopic”, “VATS”, “Bronchopulmonary Sequestration”, “PS”, and “Pulmonary Sequestration” were used. The search strategy for PubMed was as follows: (((("Thoracic Surgery, Video-Assisted"[Mesh]) OR (Video-Assisted[Title])) OR (Thoracoscopic[Title])) OR (VATS[Title])) AND (((("Bronchopulmonary Sequestration"[Mesh]) OR (Bronchopulmonary Sequestration[Title])) OR (Pulmonary Sequestration[Title])) OR (Pulmonary Sequestrations[Title])). The selection of studies was based on the abstracts and full texts. Review, letters to the editor, comments, correspondence, case reports, surgical technique notes, meeting abstracts, unpublished studies with less than 5 patients, pediatrics with PS were excluded. Finally, a total of 13 retrospective cohort studies including 350 adolescent and adult patients (range of age 14–76 years) were reviewed (Table 3).

**Table 3:** Previous reports of thoracoscopic lung surgery for adult patients with pulmonary sequestration

First author, year	No. of patients	Age, y	ILS/ELS, n	Location: left/right thorax, n	Origin of the feeding: thoracic/abdominal aorta, n	Procedure	Conversion, n to multiport or thoracotomy	Duration of operation, min	Estimated bleeding, mL	Chest tube drainage or hospital stay, d	Major complications
Kestenholz, 2006 [15]	14	20–64	13/1	6/8	10/3; 1 from right renal artery	MVATS	1 (7.1%)	133 (45–270)	200 (20–1200)	7.5 (3–13)	3 pneumonia, 1 hemothorax
Tsang, 2006 [16]	6	27–64	6/0	4/2	6/0	MVATS	0	112.8 (90–140)	283.3(100–500)	3.2	1 wound infection
Shen, 2013 [17]	25	16–62	25/0	16/9	21/2; 1 phrenic artery; 1 intercostal artery	MVATS	0	114.2 ± 31.2	228 ± 96.5	3.2 ± 1.4	0
Liu, 2013 [4]	18	15–61	16/2	14/4	15/3	MVATS	0	133.1 ± 42.3	186.1 ± 279.4	2.7 ± 0.8	1 pneumonia
Lin, 2016 [18]	26	14–68	26/0	21/5	22/3; 1 celiac trunk	MVATS	0	115.0 ± 30.4	65.0 ± 47.9	3.7 ± 1.3	1 bloody sputum
Wang, 2016 [19]	16	34.2 ± 14.0	16/0	13/3	14/2	MVATS	3 (18.8%)	122 ± 40	116 ± 90	5.9 ± 1.4	1 intractable air leakage
Lin, 2018 [20]	19	17–62	17/2	14/5	17/2	7UVATS, 12MVATS	0	(151.6 ± 43.9) vs. (173.5 ± 63.1)	(64.3 ± 62.7) vs. (87.5 ± 102.5)	(3.0 ± 1.9) vs. (4.0 ± 2.7)	1 air leakage, 1 chylothorax
Li, 2018 [21]	42	42.6 ± 11.5	42/0	32/10	35/7	1–2/3–4 ports VATS	2 (4.8%)	154 ± 52	193 ± 238	3.9 ± 1.5	1 reoperation, 3 transfusion
Li, 2019 [22]	33	23–55	33/0	19/14	24/8; 1 intercostal artery	19UVATS, 14MVATS	0	(123.5 ± 27.3) versus (128.4 ± 18.5)	100 (70–150) versus 145 (120–185)	3 (2–3) versus 3.5 (3–4)	4 chest pain/cough/hemoptysis
Wang, 2019 [23]	35	16–76	29/6	28/7	29/4; 1 subclavian artery; 1 other	MVATS	0	150 (75–300)	50 (10–600)	3 (1–10)	1 hoarseness
Li, 2020 [24]	67	15–71	65/2	48/19	57/10	21 UVATS, 46 MVATS	6 (9.0%)	126 (95–170)	100 (50–150)	4.1 ± 1.5	5 (7.5%)
Sun, 2020 [25]	24	18–65	21/3	20/4	20/3; 1 other	UVATS	0	102 (55–150)	90 (10–300)	4 (1–10)	1pneumothorax
Bishnoi, 2021 [26]	25	16–28	25/0	15/10	18/5; 2 others	NA	1 (4.0%)	179	204	3	1 reoperation, 1 air leakage
Summary	350	14–76	334 (95.4%)/16 (4.6%)	250 (71.4%)/100 (28.6%)	288 (82.3%)/52 (14.9%); 10 others (2.8%)	–	13 (3.7%)	Mean, < 180	Mean, < 300	1–13	26 (7.4%)

*ILS* intralobar pulmonary sequestration, *ELS* extralobar pulmonary sequestration, *LLL* left lower lobe, *RLL* right lower lobe, *UVATS* uniportal video-assisted thoracoscopic surgery, *MVATS* multiport video-assisted thoracoscopic surgery, *NA* not available

These studies presented 334 (95.4%) patients with IPS and 16 (4.6%) patients with EPS; whereas 250 (71.4%) and 100 (28.6%) lesions were located in the left and right thorax, respectively. In addition, 288 (82.3%) and 52 (14.9%) patients revealed aberrant feeding artery originated from the thoracic and abdominal aorta, respectively; whereas the other 10 (2.8%) showed feeding artery from the branches of the aorta including intercostal, phrenic, gastric artery. Nearly one-half of the adult PS patients were presented asymptomatically. The overall proportion of intraoperative conversion from uniportal or multiport VATS to thoracotomy was 3.7% (13/350). Moreover, the mean operation time was less than 180 min, followed by an estimated intraoperative blood loss of less than 300 mL for each patient. Major surgery-related complications such as respiratory tract infection, pneumothorax, chylothorax, and prolonged air leakage occurred in 26 (7.4%) patients. In total, only one patient (0.3%, 1/350) recorded reoperation for unknown reason. Furthermore, the postoperative chest tube duration was ranged from 1 to 13 days. The currently available evidence, although with low quality due to the limited sample size and their retrospective nature, proved the feasibility and safety of uniportal VATS anatomic lung resection for PS. As compared with the above literature, the patients in the 3D-CTA group of our study reported no conversion to thoracotomy or major bleeding, with obviously shortened operation time as compared with the control group using two-dimensional MDCT images for surgical planning. Therefore, uniportal VATS with 3D-CTA could be recommended as the optimal surgical procedure for PS.

Finally, the limitations of the present cohort study should also be indicated. Besides the small sample size and the retrospective nature of this study, the different resection range such as lobectomy versus segmentectomy also has an effect on the operation time and thorax drainage. Moreover, the accumulated experience and the learning curve regarding uniportal VATS of the surgeon also affects the surgical parameters. Considering the actually long study period (from 2011 to 2021) and the later introduction of 3D-CTA technique in our hospital, most patients in the control group performed PS resection during an early stage as compared with those in the 3D-CTA group. Therefore, the findings of the present study should be interpreted with caution. More and better evidence are warranted to further verify the advantages of 3D-CTA for precise PS resection.

## Conclusions

The current evidence confirmed the safety and advantages of uniportal VATS lung resection assisted with 3D-CTA for PS. Multicenter, well-designed trials with larger sample size are needed to validate these findings.

## Data Availability

The datasets analyzed during the current study are available from the corresponding author on reasonable request. The data generated in the review are included in this published article.

## Abbreviations

PS: Pulmonary sequestration
ILS: Intralobar pulmonary sequestration
ELS: Extralobar pulmonary sequestration
VATS: Video-assisted thoracoscopic surgery
CT: Computed tomography
3D-CTA: Three-dimensional computed tomography angiography
PICOS: Population, intervention, comparator, outcome and strategy
